# BNPLA: borated plastic for 3D-printing of thermal and cold neutron shielding

**DOI:** 10.1038/s41598-024-70030-4

**Published:** 2024-08-20

**Authors:** Simon R. Sebold, Tobias Neuwirth, Alessandro Tengattini, Robert Cubitt, Ines Gilch, Sebastian Mühlbauer, Michael Schulz

**Affiliations:** 1 Heinz Maier-Leibnitz Zentrum (MLZ), Technical University of Munich (TUM), Lichtenbergstr. 1, 85748 Garching, Germany; 2 Univ. Grenoble Alpes, Grenoble INP, CNRS, 3SR, 38000 Grenoble, France; 3 Chair of Metal Forming and Casting (utg), Technical University of Munich (TUM), Walther-Meißner-Str. 4, 85748 Garching, Germany; 4 Institut Laue-Langevin (ILL), 71 Avenue des Martyrs, 38000 Grenoble, France

**Keywords:** Fused filament fabrication, Neutron shielding, Neutron imaging, Small angle neutron scattering, Techniques and instrumentation, Techniques and instrumentation

## Abstract

3D printing technologies such as fused filament fabrication (FFF) offer great opportunities to enable the fabrication of complex geometries without access to a workshop or knowledge of machining. By adding filler materials to the raw filaments used for FFF, the material properties of the plastic can be adapted. With the addition of neutron absorbing particles, filaments can be created that enable 3D printing of neutron shielding with arbitrary geometry. Two materials for FFF are presented with different mixing ratios of hexagonal Boron nitride (h-BN) and Polylactic acid (PLA). BNPLA25 with 25 %wt h-BN and BNPLA35 with 35 %wt h-BN are compared to the commercially available Addbor N25 material. To qualify the applicability of BNPLA25 and BNPLA35 as shielding material for neutron instrumentation, such as neutron imaging, we investigated the overall neutron attenuation, the influence of non-optimized print settings, as well as characterized the incoherent neutron scattering and the microstructure using neutron imaging, and time-of-flight small-angle-neutron-scattering. Finally, the tensile strength of the material was determined in standardized tensile tests. The measured neutron attenuation shows excellent agreement with analytical calculations, thus validating both the material composition and the calculation method. Approximately 6 mm (8 mm) BNPLA35 are needed for $1\times 10^{-3}$ transmission of a cold (thermal) neutron beam. Lack of extrusion due to suboptimal print settings can be compensated by increased thickness, clearly visible defects can be mitigated by 11–18% increase in thickness. Incoherent scattering is shown to be strongly reduced compared to pure PLA. The tensile strength of the material is shown not to be impacted by the h-BN filler. The good agreement between the measured attenuation and calculation, combined with the adoption of safety factor enables the quick and easy development as well as the performance estimation of shielding components. BNPLA is uniquely suited for 3D printing neutron shielding because of the combination of non-abrasive h-BN particles in standard PLA, which results in a filament that can be printed with almost any off-the-shelf printer and virtually no prior experience in 3D printing. This mitigates the slightly lower attenuation observed as compared to filaments containing ${\hbox {B}_{4}}\hbox {C}$, which is highly abrasive and requires extensive additive manufacturing experience.

## Introduction

Effective shielding of cold and thermal neutrons is essential for neutron instrumentation to limit activation of instrument components, sample environment and areas of samples not directly examined, as well as increase collimation and reduce background radiation. This is especially relevant in neutron imaging as the neutron flux is usually high because of the comparatively large beam size throughout the experimental setup. Several options are available to shield samples and components from activation. Cadmium (Cd) metal sheets and borated plastics are widely used. Cd, however, is toxic^1^, quite soft at room temperature, and produces high-energy gamma radiation upon neutron capture^2^. Its toxicity and softness limit the possibility of machining custom parts from Cd. It is, therefore, not ideal as a general-purpose neutron shielding material. Borated plastics are usually made from a plastic compound with a Boron containing filler material added, for example, commonly available rubber mixed with Boron carbide ${\hbox {B}_{4}}\hbox {C}$^3^ or Polyethylene (PE) with Boron trioxide $(\hbox {B}_{2}\hbox {O}_{3})$^4^. However, such materials are often only available as pre-product and require further machining, which in turn requires a workshop and can be time and resource-intensive. To rapidly manufacture custom parts from thermoplastic materials fused filament fabrication (FFF) 3D printing is a quickly developing option. In FFF, a thin line of plastic is extruded through a small-diameter heated nozzle. By moving the extruder, arbitrarily shaped layers can be constructed and subsequently stacked on top of a previous layer along the so-called build direction. Given a suitable plastic compound extruded to a standard-size filament, it is possible to manufacture almost arbitrary parts within a few hours. By using a thermoplastic material mixed with a boron-containing filler, it is possible to create custom shielding solutions.

There is commercially available borated filament (Addbor N25^5^) based on Polyamide (PA) with ${\hbox {B}_{4}}\hbox {C}$ filler, which shows excellent shielding capabilities due to the high Boron content of 25 %wt ${\hbox {B}_{4}}\hbox {C}$^6^. However, due to the abrasive nature of the ${\hbox {B}_{4}}\hbox {C}$ particles^7^, hardened printer nozzles^8^ are required. Moreover, PA filaments tend to be difficult to print and require additional preparation for optimal printing results, such as drying the filament and preparation of the print-bed with glue stick^9^. Additionally, it cannot be machined easily after printing due to the abrasive ${\hbox {B}_{4}}\hbox {C}$ particles. Its high price of ca. 1000 €/kg^10^ also limits its applicability for large bulk shielding solutions.

Woosley et al.^11^ showed, that Acrylonitrile butadiene styrene (ABS) can be dissolved in Acetone and mixed with h-BN and subsequently extruded to standard size filament. However, dissolving large quantities of ABS in Acetone is not feasible, and ABS is not ideal as it emits toxic fumes while printing^12^ and requires similar preparations for printing as PA^9^. Wu et al.^13^ used a scalable process for manufacturing a mixture of Polyether ether ketone (PEEK) and ${\hbox {B}_{4}}\hbox {C}$ by heating and extruding the composite powder to standard sized filaments. PEEK shows high mechanical strength but is very challenging to print, and adding ${\hbox {B}_{4}}\hbox {C}$ as filler imposes further limitations, as explained before. Knott et al.^14^ mixed h-BN at a fraction of 20 %wt with Polyurethane (PU). However, PU is quite uncommon in FFF, and commonly available printers are therefore not optimized for it.

An ideal borated filament for FFF of bulk neutron shielding would fulfill the following properties:

- Easy to print with standard FFF printers
- Affordable ($$<{100}$$ €$${\hbox {kg}^{-1}}$$)
- Manufacturing on large scales possible ($>{100}\,\hbox {kg}$)
- High neutron attenuation (max. few $\hbox {mm}$ thickness needed for $<10^{-2}$ transmission)
- Additional machining after printing possible

Boron nitride in its hexagonal (graphite-like) form (h-BN) has lower boron content than ${\hbox {B}_{4}}\hbox {C}$ but is non-abrasive and available in large quantities with known particle size. The most common plastic for FFF 3D printing is PLA, which can be printed with basically all off-the-shelf printers. By mixing PLA with different mass fractions of h-BN, it was therefore possible to manufacture a new neutron shielding material that is effective and can be printed without much prior experience in 3D printing. We chose a mass fraction of 25 %wt h-BN in PLA for a first batch, in the following referred to as BNPLA25, and 35 %wt for a second batch (BNPLA35).

To qualify the applicability of BNPLA25 and BNPLA35 as shielding material for neutron instrumentation, especially neutron imaging instrumentation, we investigated neutron attenuation, the influence of non-optimized print settings, incoherent neutron scattering as well as the microstructure, and finally, the mechanical strength of the material.

## Results

The results presented in the following sections were achieved using multiple methods. Neutron imaging at the NeXT beamline of ILL^15^ was employed to determine the neutron attenuation of BNPLA25, BNPLA35, and Addbor N25 as well as the influence of print settings and estimate the incoherent scattering of the material. The microstructure, wavelength-dependent neutron attenuation as well as the incoherent neutron scattering of BNPLA35 was determined with time-of-flight (ToF) small-angle-neutron-scattering (SANS) measurements at the D33 beamline of ILL^16^. Finally, the tensile properties of the compounds were determined using a standard tensile rig. A more detailed explanation of the measurements and calculation of the neutron interaction with the material is given in the methods section.

### Attenuation

**Figure 1:**
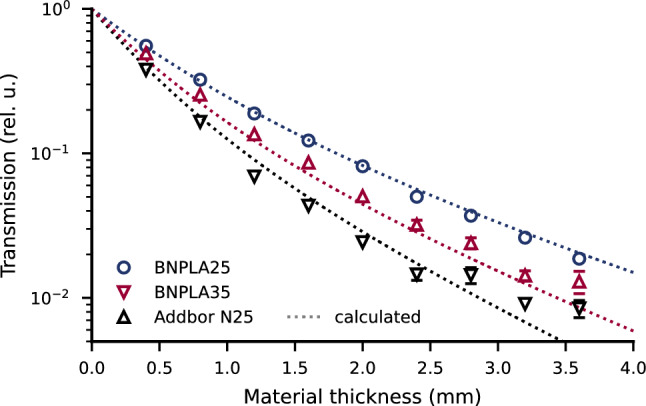
Neutron transmission over material thickness. Comparison of PLA with 25 %wt h-BN (B25h, dark blue circles) to PLA with 35 %wt h-BN (B35h, red squares) and Addbor N25 (A25h, black triangle). The measured transmission matches the expected calculated trend (dotted lines) well for high transmission. Below transmission values of approx. $10^{-2}$ the data deviate from the expected values.

To evaluate the neutron attenuation of the material, transmission measurements of BNPLA25, BNPLA35, and Addbor N25 were performed using neutron imaging at the NeXT beamline at the ILL^15^. The samples used were 3D-printed step-wedge samples (see Figs. 12a,b), listed in Table 5. The measured transmission over thickness is shown in Fig. 1 for BNPLA25 (dark blue circles), BNPLA35 (red tip-up triangles), and Addbor N25 (black tip-down triangles). The BNPLA25 and Addbor N25 samples were placed ${150}\,\hbox {mm}$ in front of the detector. The BNPLA35 samples were placed close to the detector. If no error bars are given, the uncertainties in thickness and the standard deviation of the transmission values across the evaluated areas lie within the area of the markers. By assuming material compositions and densities as summarized in Tables 3 and 4, the expected wavelength-dependent transmission is calculated as detailed in the methods section and adapted for the specific spectrum of NeXT^15^ using Eq. 4. The resulting calculated transmission is shown as dotted lines in Fig. 1.

The measured transmission data match the calculated transmission well in trend and absolute values. The transmission decreases with increasing Boron content, with BNPLA25 (dark blue) showing the lowest, BNPLA35 (red) intermediate, and Addbor N25 (black) the highest attenuation, respectively. Values below approximately $10^{-2}$ deviate from the expected transmission due to so-called “beam starvation” in the strongly absorbing areas and the resulting increased contribution of noise to the signal. The transmission of BNPLA35 is consistently slightly higher than predicted by calculation. This can be attributed to incoherently scattered neutrons being detected in the transmission as the BNPLA35 sample was placed right in front of the detector, as detailed later. The fraction of incoherently scattered and subsequently detected neutrons is significantly lower for BNPLA25 and Addbor N25 because those samples were placed 150 $\hbox {mm}$ away from the detector.

**Figure 2:**
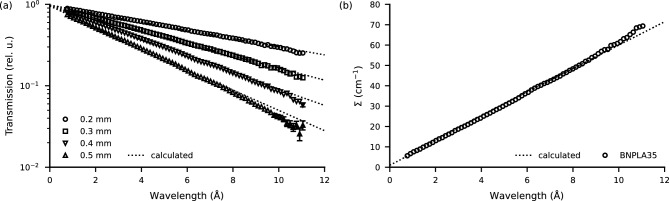
(**a**) Wavelength-dependent neutron transmission of BNPLA35 for material thicknesses of 0.2 $\hbox {mm}$ (circles), 0.3 $\hbox {mm}$ (squares), 0.4$\,\hbox {mm}$ (tip-down triangles) and 0.5 $\hbox {mm}$ (tip-up triangles) as well as the corresponding calculated transmission (dotted lines). (**b**) Linear attenuation coefficient $\Sigma (\lambda )$ calculated from the transmission in (**a**) averaged over the different thicknesses (circles) compared to the calculated coefficient (dotted line).

For BNPLA35, the wavelength-dependent neutron transmission was measured using ToF at D33 of ILL^16^ for four samples of different thicknesses. The detector was placed 13 $\hbox {m}$ behind the sample, strongly reducing the contribution of scattering to the transmission signal. Figure 2a shows the transmission over wavelength for a thickness of 0.2 $\hbox {mm}$ (circles), 0.3 $\hbox {mm}$ (squares), 0.4 $\hbox {mm}$ (tip-down triangles) and 0.5 $\hbox {mm}$ (tip-up triangles) as well as the calculated transmission as dotted lines. The uncertainties lie within the size of the markers unless otherwise indicated. Due to the dominant absorption cross-section of h-BN, the main trend of the transmission in logarithmic representation is linear in wavelength. The slope scales with material thickness. Using Eq. 5, the wavelength-dependent linear attenuation coefficient $\Sigma (\lambda )$ can be obtained for each sample independent of the thickness. The subsequently averaged coefficient is shown in Fig. 2b and matches the calculated coefficient very well, except for a systematically lower transmission for wavelengths above approximately 9 Å. At approximately 6.5 Å a slight bump of the last bragg-edge of h-BN, as shown in Fig. 10a, is visible. This deviation above 9 Å is probably due to the average functional group approximation (AFGA)^17^ used in the model for inelastic scattering deviating from the actual cross-section.

Table 1 shows a list of calculated thicknesses of material needed to reach a given neutron attenuation for a cold (NeXT) and a thermal (Maxwellian with peak at 1.8 Å) spectrum.

**Table 1:** Comparison of the calculated thickness of shielding material needed to achieve given orders of magnitude of neutron transmission in the cold neutron spectrum of NeXt^15^ and a thermal Maxwellian spectrum with peak at 1.8 Å.

Transmission (rel. u.)	Thickness BNPLA25 (mm)		Thickness BNPLA35 (mm)	
	Cold	Thermal	Cold	Thermal
$10^{-2}$	4.54	6.47	3.44	4.93
$10^{-3}$	7.94	10.6	6.04	8.11
$10^{-4}$	11.7	15.2	8.92	11.6

### Influence of print settings

**Figure 3:**
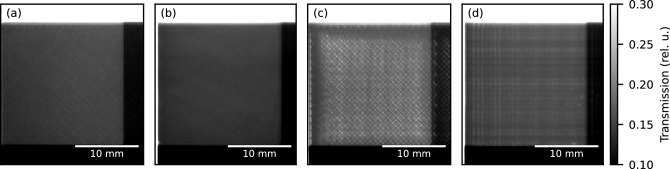
Transmission maps for different print settings of the 1.6 $\hbox {mm}$ thick area of the step-wedges. The samples were placed directly in front of the detector. Optimized print settings in horizontal (**a**) and vertical (**b**) build directions show homogeneous attenuation as compared to non-optimized print settings in horizontal (**c**) and vertical (**d**) build directions with locally varying attenuation.

**Figure 4:**
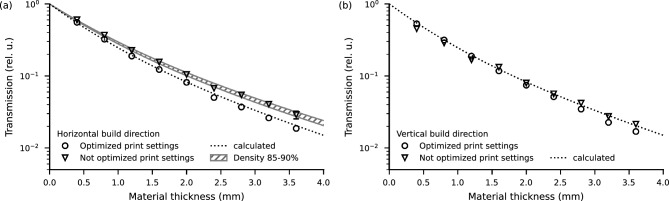
Optimized (circular markers) and not optimized (square markers) print settings for horizontal (**a**) and vertical (**b**) build direction. The transmission of the material printed with not optimized print settings is generally higher than optimized, except the thinnest three steps for the vertical build direction show lower transmission than calculated. The hatched area shows the calculated transmission for a reduced BNPLA25 density of 85–90%.

The close match of measured and calculated transmission shows that it is possible to print with very little inner porosity and basically reach bulk densities. However, suboptimal print settings will lead to defects and inconsistencies in the printed part, which can often even be spotted by the naked eye. Furthermore, the layering of FFF perpendicular to the build direction is, in general, easily distinguishable. It is, therefore, of interest to characterize the influence of print settings and build direction on neutron transmission. By intentionally choosing unoptimized print settings that show a visible lack of extrusion as a worst-case example, a lower limit on achievable bulk density due to non-visible defects can be estimated. Figure 4 shows the transmission averaged over a 20x20 $\hbox {mm}^{2}$ area with 1.6 $\hbox {mm}$ thickness of step-wedges, printed with optimized (Fig. 3a,b) and not optimized print settings (Fig. 3c,d) for each horizontal (Fig. 3a,c) and vertical build direction (Fig. 3b,d).

For the optimized print-settings the whole $20\times 20\,\hbox {mm}^{2}$ area shows homogeneous transmission of 12.3(5)% for the horizontal (Fig. 3a) and 11.7(4)% for the vertical build direction (Fig. 3b). In the transmission map of the horizontally built step-wedge, the lines of the FFF extrusions can be seen faintly, whereas the vertical build direction shows a slight decrease of transmission towards the left edge. The transmission maps of the step-wedges printed with not optimized settings can clearly be distinguished between horizontal (Fig. 3c) and vertical (Fig. 3d) build direction. For the horizontal build direction, the hatched pattern of alternating ${45}\,^{\circ }$ extrusion lines is visible with decreasing transmission towards the top and right edges and total average transmission of 15.3(10)%. The vertical build direction exhibits vertical stripes in a regular pattern on the right edge and irregular horizontal stripes across the whole area. The left edge attenuates slightly more, and the average transmission is 13.2(4)%.

The trend of higher attenuation towards the left edge for the vertical prints (Fig. 3b,d) and towards the top and left for the horizontal not optimized print (Fig. 3c) can be attributed to the printer depositing slightly more material upon change of direction. The effect can be mitigated by adjusting the print settings and is, therefore, more pronounced in the not optimized prints.

The irregular horizontal lines in the not optimized vertical print are most likely due to inconsistent extrusion. The vertical lines result from oscillations in the mechanical drive train of the print head due to the sudden change of momentum on the corner, leading to inconsistencies in thickness. The difference in average transmission between optimized and non-optimized print settings is due to more material being deposited in the bulk of the step-wedge after optimization. The optimized vertical sample (Fig. 3b) has slightly higher attenuation than its horizontally printed counterpart (Fig. 3a). This is likely due to common FFF printers having slightly higher precision parallel to the build direction than perpendicular to it. As already very minute offsets in the amount of extruded material will change the thickness of the extruded line perpendicular to the build direction, but the nozzle surface limits the thickness of the extrusion parallel to the build direction.

Figure 4 shows a comparison of the transmission depending on the thickness of horizontally (Fig. 4a) and vertically printed step-wedges (Fig. 4a) with optimized (circular markers) and not optimized print settings (triangle markers). In the horizontally printed samples, see Fig. 4a, not optimized print settings lead to an overall reduction in attenuation corresponding to a mean density reduction due to insufficient extrusion and, therefore, air gaps. For vertically printed samples, shown in 4b, the lowest three thickness steps show higher attenuation than expected, which is most likely due to thicker extrusion perpendicular to the build direction, as mentioned before for the vertically printed transmission maps. The smallest three thickness values are one, two, and three times the nozzle diameter. They are printed as one, two, and three solid lines next to each other and, therefore, suffer from the aforementioned over-extrusion perpendicular to the build direction. Moving to thicker steps, this over-extrusion is no longer present when the print is performed as two perimeter lines and a ${45}^{\circ }$ hatched fill pattern. Then, the non-optimized extrusion multiplier causes too little material to be deposited and subsequently causes gradually less than expected attenuation for thicker steps.

To conclude, suboptimal print settings reduce the material’s attenuation by locally reducing the effective density of Boron. In the horizontal build direction an approximate reduction of bulk density to 85–90% was observed, see Fig. 4a. However, the effects are not drastic and can be mitigated by considering safety margins in the thickness of the material, in this case, a factor of 1.11 to 1.18.

### Incoherent scattering

**Figure 5:**
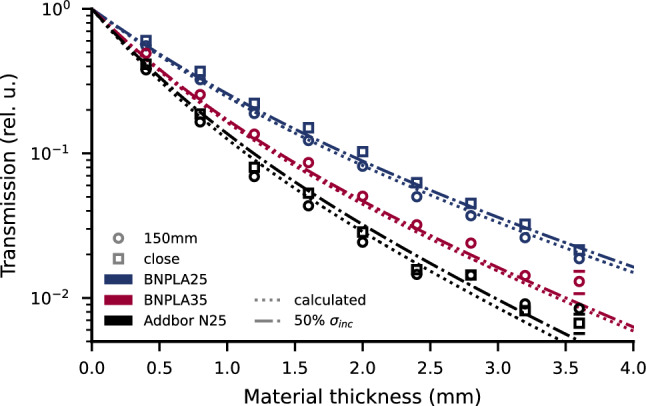
Transmission for a given material thickness at 150 $\hbox {mm}$ (circular markers) from the detector compared to directly in front of the detector (square markers) for BNPLA25 (blue), BNPLA35 and Addbor N25 (black). The dotted lines show the calculated transmission when assuming none of the neutrons interacting with the material are detected and the dash-dotted lines show the expected transmission if 50% of the neutrons incoherently scattered are still detected.

The high content of Hydrogen in the plastic raises the question of the level of incoherent scattering and the resulting contribution to undesired background signal in measurements. When comparing the transmission values from the same sample at different distances to the detector, a very rough estimate of the amount of incoherent scattering can be obtained. If the sample is placed directly in front of the detector, approximately half of the solid angle, as seen from the point of the interaction of the neutron with the material, is covered by the detector. Therefore, approximately half of the neutrons that scatter incoherently are still detected and, therefore, increase the measured transmission. By placing the step-wedges 150 $\hbox {mm}$ away from the detector, the probability of incoherently scattered neutrons hitting the detector is significantly reduced. Figure 5 shows the transmission of BNPLA25, BNPLA35, and Addbor N25 each directly in front of the detector and 150 $\hbox {mm}$ away. The transmission of BNPLA25 (dark blue) is higher if the step-wedge is close to the detector (square markers) as compared to the measurement with the step-wedge at 150 $\hbox {mm}$ from the detector (round markers). For Addbor N25 (black), the same trend of higher transmission with the sample close to the detector can be seen. The transmission of BNPLA35 (red) was only measured close to the detector.

If the sample exhibits a significant amount of neutron scattering, more scattered neutrons are detected when placing the sample closer to the detector due to the increase in the solid angle covered by the detector. This leads to an increase in the measured transmission. As an upper boundary we can assume half of the solid angle covered by the detector when placing the sample close to the detector, thus 50% of the scattered neutrons are detected. This can be modeled by reducing the incoherent neutron cross-section to 50%. Figure 5 therefore shows the calculated transmission with the incoherent scattering cross-section reduced by 50% (dash-dotted lines) as well as the calculated transmission (dotted lines) as shown before in Fig. 1. This is obviously a crude estimation, but the resulting increase in calculated transmission matches the measured transmissions at close distances to the detector reasonably well for all materials, including BNPLA35. The scattering under small angles of the other modes is not considered here but is expected to contribute to the increase in the measured transmission as well. The influence of the incoherent scattering cross-section is similar in Addbor N25 and BNPLA25 due to the similar volume fractions of filler in plastic and lower for BNPLA35 due to the high volume fraction of h-BN, see Table 4.

**Figure 6:**
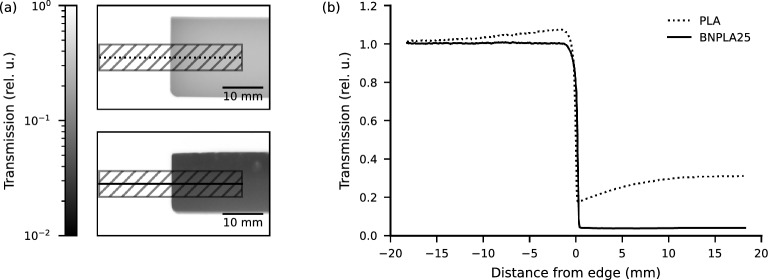
(**a**) Transmission maps for two 8 $\hbox {mm}$ thick cast blocks of pure PLA (upper) and BNPLA25 (lower). The direction of the line profiles shown in (**b**) is indicated as a dotted (PLA) and continuous (BNPLA25) line﻿ taking the mean over the hatched area. (**b**) Line profiles of the transmission as shown in (**a**). Pure PLA (dotted line) shows higher transmission than unity directly next to the block, starting at approximately 1.1 directly next to the edge and still slightly higher than 1 at − 20 $\hbox {mm}$ distance from the edge. The transmission in the material of approximately 0.3 is distributed non-uniformly. For BNPLA25 (continuous line), the transmission does not exceed unity outside the sample (distance from edge $<{0}\hbox {mm}$) and drops close to zero in the material (distance from edge $>{0}\hbox {mm}$).

To compare the incoherent scattering of pure PLA to BNPLA25, radiographs of cast blocks with 8 $\hbox {mm}$ thickness were taken. Figure 6a shows the transmission maps of the cast blocks, PLA in the upper and BNPLA25 in the lower panel. Line profiles of the transmission along the continuous lines in Fig. 6a, averaged across the hatched area, are shown in Fig. 6b. They reveal transmission values above 1 next to the block of PLA (dotted line). With transmission being the ratio of measured intensity with a sample to the intensity measured without a sample, the signal on the detector in the area next to the block of PLA indicates a significant amount of neutrons being scattered incoherently. This agrees with the profile within the block of PLA, showing lower transmission towards the edge as there is only scattering material to one side. The transmission next to the block of BNPLA25 is 1 and steeply drops close to zero within the material. Thus indicating that the amount of incoherently scattered neutrons from borated PLA is significantly reduced compared to regular PLA.

The transmission measurements at different distances to the detector indicate incoherent scattering proportional to the volume fraction of plastic in BNPLA25, BNPLA35, and Addbor N25. The amount of scattering is, however, greatly reduced compared to pure PLA as shown in Fig 6b due to the additional absorption of scattered radiation by Boron. Next to the block of BNPLA25 no scattered neutrons are detected, but the change in measured transmission when changing the distance to the detector indicates the presence of scattering. When observing the area next to the sample, mostly neutrons scattered under wide angles are detected, but when observing the transmission value at different distances, mostly small scattering angles contribute to a change in the signal. Additionally, large scattering angles are strongly suppressed in flat geometries as the scattered neutrons need to propagate through significantly more material with increasing scattering angles, therefore explaining the lack of scattered neutrons next to the sample.

However, this is only a simple estimation of the contribution of incoherent scattering aimed to highlight how this has no drastic influence on neutron imaging applications and needs to be further investigated, especially with respect to its applicability in neutron scattering instrumentation.

**Figure 7:**
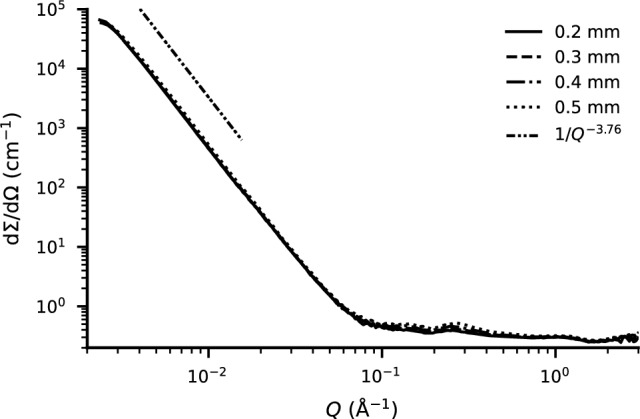
Small angle neutron scattering for different material thicknesses of BNPLA35.

The SANS measurements shown in Fig. 7 indicate a total isotropic, thus including incoherent, scattering cross-section of $\Sigma = {3.8\pm 0.4}\,\hbox {cm}^{-1}$ when taking the average of the scattering coefficient $d\Sigma /d\Omega$ over the range of Q = 0.4to 2.4 Å${}^{-1}$ and integrating over the whole solid-angle, i.e. multiplying with $\mathrm {4\pi }$ by assuming isotropic scattering.

### Microstructure

The scattering of the material was measured using small-angle neutron scattering (SANS) at the D33 beamline of ILL^16^. For this, four disks with a diameter of 25 $\hbox {mm}$ and different thicknesses of BNPLA35 were printed, as detailed in Table 6. The macroscopic scattering cross-section ($\textrm{d}\Sigma /\textrm{d}\Omega$) was obtained by standard data treatment of ToF SANS data and is shown in Fig. 7 for the 4 different samples after correction for the different thicknesses of 0.2 $\hbox {mm}$, 0.3 $\hbox {mm}$, 0.4 $\hbox {mm}$ and 0.5 $\hbox {mm}$. By linear fitting $ln(\textrm{d}\Sigma /\textrm{d}\Omega )$ over *ln*(*Q*) between Q = 0.0035 Å${}^{-1}$ and 0.016 Å${}^{-1}$ the Porod slope *n* was determined for each thickness, which averages to $n = {-3.760(5)}$, illustrated by the black dashed line in Fig. 7. This indicates scattering of large particles with a rough surface, which can be attributed to the $\upmu \hbox {m}$ sized h-BN particles in the polymer. For high *Q*, the scattering cross-section levels off to a constant value representing the incoherent scattering arising from the hydrogen-rich polymer. This behavior is similar to Addbor N25 as described in^6^.

### Tensile properties

**Figure 8:**
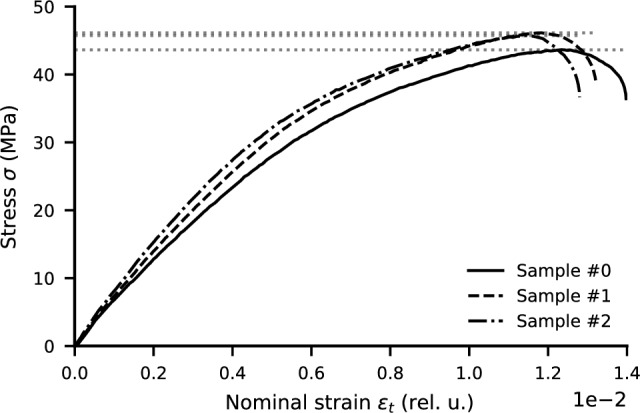
Stress over nominal strain for three samples resulting from tensile tests with a pull rate of 1  $\hbox {mm}\,\hbox {min}^{1}$ until break. The dotted lines indicate the maximum tensile stress reached of each sample.

The tensile properties of BNPLA35 were measured according to DIN EN ISO 527-1 using three samples printed with the build direction perpendicular to the tensile loading direction. The area of the tapered region of the sample perpendicular to the pulling direction is 2x2 $\hbox {mm}^{2}$. The measurements were performed using a standard tensile rig without an extensometer, therefore using nominal strain $\epsilon _t = \Delta L / L_0$, with $L_0 = {20}\,\hbox {mm}$ being the distance of the mounting grips at the start of the pull. Three pulls were performed until breaking of the sample with a pull rate of 1 $\hbox {mm}\,\hbox {min}^{1}$. The resulting stress-strain curves are shown in Fig. 8, resulting in a maximum tensile strength $\sigma _{max}$ of ${45.2(11)}\,\hbox {MPa}$. This is identical to the tensile strength of the raw material used to fabricate BNPLA35 as specified by the manufacturer^18^. The obtained results for BNPLA35 show, therefore, no significant reduction in tensile strength by adding h-BN as filler (Table 2). The tensile strength of BNPLA35 is in the medium range of commercially available PLA, as typical values for PLA from different manufacturers vary between 35 $\hbox {MPa}$ and 65 $\hbox {MPa}$^9^.

**Table 2:** Tensile strength for the three tensile test samples.

Sample	Tensile strength (MPa)
#0	43.6
#1	46.1
#2	45.7
Avg.	45.2 (1.1)

## Discussion

The material composition of Polylactic acid (PLA) with both 25 %wt (BNPLA25) and 35 %wt h-BN (BNPLA35) proved to produce a neutron shielding material that can be printed with low effort on off-the-shelf FFF printers. Due to the availability of both PLA and h-BN as well as the non-abrasive nature of h-BN, which limits undue wear on the fabrication hardware for filament extrusion, the cost of this material is comparably low with approximately 50–60 €${\hbox {kg}^{-1}}$. h-BN with natural isotopic abundance was chosen due to the aforementioned availability in large quantities and low price. Using h-BN with pure ${}^{10}\text {B}$ (enriched h-BN) would decrease the required thickness for given transmissions significantly as the linear absorption coefficient for enriched h-BN is roughly five times higher than h-BN with naturally abundant ${}^{10}\text {B}$^19^. However, for conventional bulk shielding applications we believe the benefit of low cost and easy accessibility outweigh the performance gain. Especially since it can be compensated by only a few $\hbox {mm}$ of additional shielding.

The expected neutron transmission of the material was calculated using the Monte-Carlo based simulation tool NCrystal^20–22^ and compared to measurements. The transmission of BNPLA25, BNPLA35, and the commercially available Addbor N25 show good agreement between expected calculated transmission and measured values for a polychromatic cold neutron beam at different thicknesses. For BNPLA35, wavelength-dependent transmission using ToF measurements for different material thicknesses was used to obtain the wavelength-dependent linear attenuation coefficient, which is in good agreement with the calculation as well. Approximately ${6}\,\hbox {mm}$ (${8}\hbox {mm}$) BNPLA35 are needed for $1\,\times \,10^{-3}$ transmission of a cold (thermal) neutron beam. The attenuation of BNPLA25 is slightly lower with ${8}\,\hbox {mm} ({11}\,\hbox {mm}$) needed for $1\,\times \,10^{-3}$ transmission of a cold (thermal) neutron beam. The attenuation of Addbor N25 is higher than BNPLA due to the higher Boron content of the ${\hbox {B}_{4}}\hbox {C}$ filler.

BNPLA is easy to print with standard settings for PLA. Depending on the default settings of the printer and slicer software, optimization of the print settings may be necessary to achieve properties similar to those of bulk material. Still, limited print quality, and especially low extrusion, can be compensated by simply increasing the thickness of the shielding material in many cases. In our testing, a safety factor of approximately 1.11 to 1.18 in thickness was sufficient to compensate for a clearly visible lack of extrusion.

The amount of incoherently scattered neutrons was estimated from transmission measurements and shown to be significantly reduced compared to pure PLA. Neutron transmission measurements taken with the sample at different distances from the detector indicate scattering proportional to the volume fraction of plastic under small angles. The SANS measurements indicate a total isotropic scattering cross-section of $\Sigma = {3.8\pm 0.4}\,\hbox {cm}^{-1}$ which can be seen as an upper boundary for the incoherent scattering cross-section. Due to the significant reduction of scattered neutrons and the suppression of large scattering angles, we expect incoherent scattering of BNPLA to not negatively impact the background signal in neutron imaging applications.

The microstructure was investigated with SANS measurements. Neutron scattering under small angles follows the Porod law with a slope of $n={-3.76}$, indicating large particles with slight surface roughness. This matches the expected behavior of large (median size 3.5 $\upmu \hbox {m}$^23^) h-BN particles in the plastic matrix, and BNPLA shows similar behavior as Addbor N25^6^.

The tensile strength of the material is similar to typical PLA and identical to the manufacturer specifications of the raw PLA used for the fabrication. This shows that the mechanical properties are not reduced due to the added h-BN.

As most of the volume of BNPLA is PLA, we expect the material properties to be very similar to those of raw PLA and thus in general behave as those of typical thermoplastic material. We, therefore, expect heat deflection temperature (ISO 75-1) of BNPLA to be close to that of the used raw PLA, which is approximately ${60}\,{^{\circ }\hbox {C}}$^18^, as well as embrittlement at cryogenic temperatures as shown for pure PLA by Liu et al.^24^. Very preliminary tests showed significant outgassing when placed in vacuum. Further measurements need to be conducted if this can be mitigated by surface treatment as described by e.g. Heikkinen et al.^25^and Rivera et al.^26^. PLA is biodegradable, but the decomposition in aqueous solutions takes in the range of years^27^. Exposure to Acetone should be avoided as it is soluble in organic solvents^28^. However, from our experience, the material is stable under brief exposure to Isopropyl Alcohol.

## Conclusions

**Figure 9:**
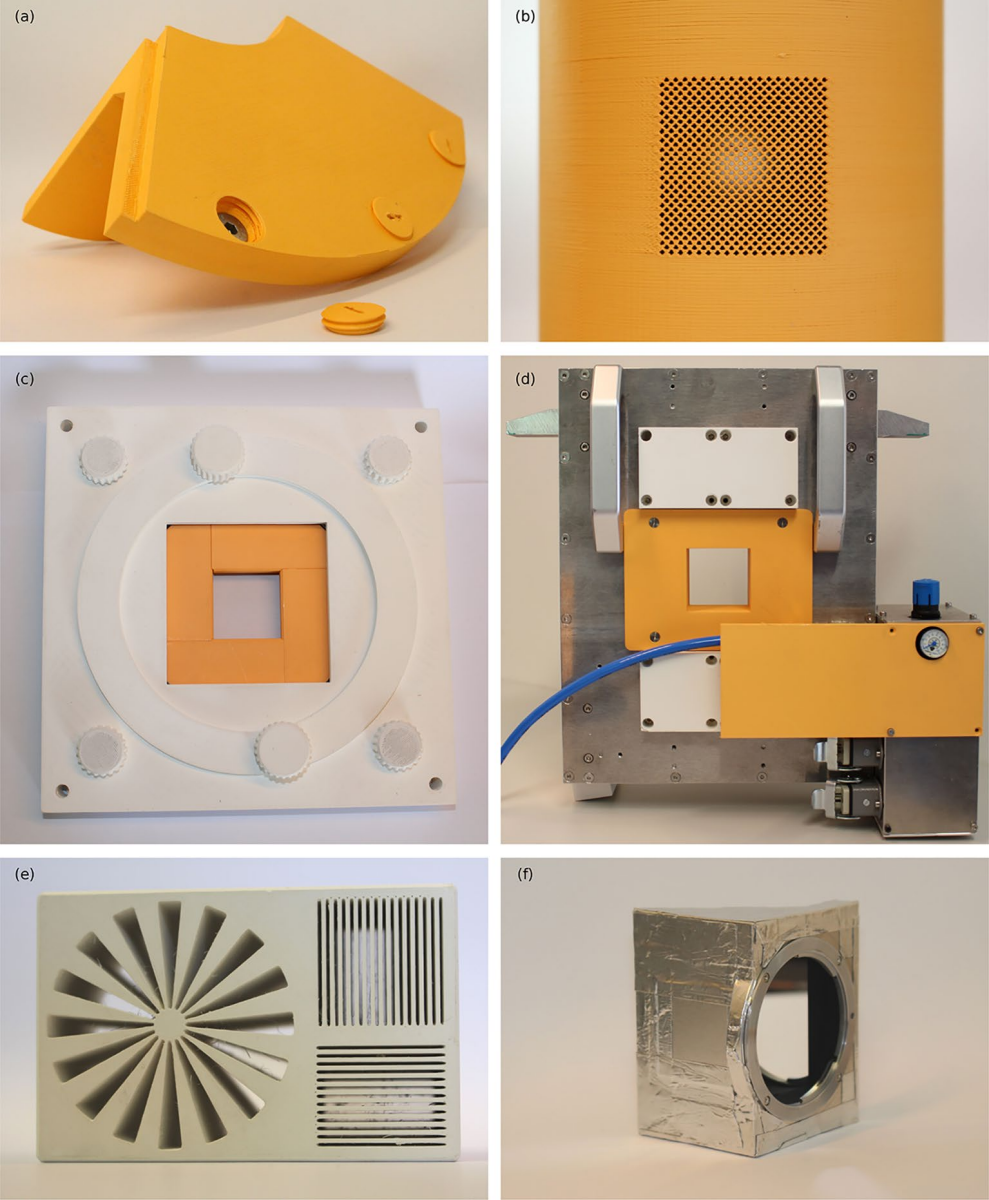
Examples for the application of BNPLA. Orange parts were printed from BNPLA35 and white parts from BNPLA25. (**a**) Shielding part for a goniometer with caps to cover stainless steel bolts. (**b**) Channel array with 684 $1\,\times \,1\hbox {mm}^{2}$ channels in the cylindrical absorber of a chopper system for the NECTAR instrument at FRM II. (**c**) Adjustable mechanical iris aperture to limit the neutron beam. (**d**) Shielding components to reduce neutron activation for a magnetic yoke sample environment. (**e**) Resolution test target for imaging with fast neutron radiation. (**f**) Scintillator and mirror holder of a neutron microscope printed from BNPLA35 and subsequently painted and covered in Aluminum tape.

An easily printable, affordable, and versatile shielding material for thermal and cold neutrons was developed. We were able to show good agreement between the transmission measurements and the calculated values. Showing that the shielding performance can be predicted reliably with simple analytical calculations. This verifies the material composition and expected shielding performance of BNPLA and enables easy and reliable dimensioning of neutron shielding using this material. Furthermore, the good agreement with calculation and thus the nominal material density shows that sufficient bulk densities can be achieved by FFF 3D printing.

In the future, a more in-depth characterization of the mechanical properties are planned, as well as studies on radiation hardness, especially possible embrittlement from high neutron doses. To ensure no interference with measurements for other neutron methods by introducing additional background when employing the shielding material close to the beam when employed, for example, as a sample holder, further measurements need to be conducted. The incoherent scattering could, for example, be characterized in a powder diffractometer.

The low cost per  $\hbox {kg}$ allows for printing large structures that would traditionally be manufactured from plate stock of, for example, borated PE. In addition to replacing work requiring a workshop and large machines, 3D printing offers unique opportunities that would otherwise not be feasible. For example, shielding parts were built and tested, tailored to the stages of the neutron grating interferometer setup^29^ of the ANTARES beamline of FRM II^30,31^, including threaded bolt covers to be able to use standard stainless steel bolts close to the beam as shown in Fig. 9a. As well as shielding parts for a magnetic yoke sample environment (see Fig. 9d) and even functional components such as an adjustable mechanical iris aperture to limit the neutron beam, shown in Fig. 9c, can be fully printed. With FFF, rapid manufacturing of components on short notice, such as arbitrarily shaped apertures tailored to the requirements of an experiment or shielding of parts of a sample, is possible. By printing parts deployed close to the beam directly from BNPLA, the need for additional shielding can be eliminated, which is especially useful due to the negligible activation of BNPLA^32^. This was successfully adopted for the scintillator and mirror holder of a neutron microscope setup where the constraints of the setup do not allow for shielding. Figure 9f shows the resulting part. The inside was painted matte black to limit optical reflection and scattering and the outside covered in Aluminum tape to shield from surrounding light. Even geometries not manufacturable without significant effort are easily printable, such as an array of $1\,\times \,1\hbox {mm}^{2}$ channels for the absorbing part of a chopper system designed for the NECTAR instrument of FRM II^33,34^ depicted in Fig. 9b. The large Hydrogen content enables moderation and subsequent absorption in Boron of fast neutrons as was used for a resolution test sample for the instrument NECTAR shown in Fig 9e.

To conclude, the material is uniquely suited for multipurpose shielding applications, especially to shield instrument components and manufacture sample holders or custom apertures, due to its ease of use, high neutron attenuation, and compatibility with off-the-shelf 3D printers.

## Methods

### Calculation of neutron shielding properties

Neutron transmission *T* through matter of a given thickness *t* is described by the Beer–Lambert law^35–37^:

1 $$\begin{aligned} T = exp\left\{ -\Sigma \left( \lambda \right) \cdot t\right\} , \end{aligned}$$

with the linear attenuation coefficient $\Sigma$, which depends on the material and neutron wavelength $\lambda$. This assumes that no neutron interacting with the material is being detected, i.e., the neutron is either absorbed in the material or scattered to a solid angle not covered by the neutron detector. The linear attenuation coefficient relates to the total microscopic cross-section $\sigma _{t}$ as such:

2 $$\begin{aligned} \Sigma \left( \lambda \right) = \frac{\rho }{M}N_A\sigma _{t}\left( \lambda \right) , \end{aligned}$$

with the density of the material $\rho$, the molar mass *M* and the Avogadro constant $N_A$^38^. Here, $\sigma _{t}$ is the average over the contributions of the isotope-specific cross-sections according to the isotopic composition of the material. In the following, natural isotopic abundance will be assumed for all calculations. The microscopic cross-section for thermal and cold neutrons in matter can be described by separate absorption ($\sigma _{abs}$), elastic (incoherent $\sigma _{inc}$ and coherent $\sigma _{coh}$) scattering as well as inelastic scattering ($\sigma _{inel}$) cross-sections, as given in Eq. 3.

3 $$\begin{aligned} \sigma \left( \lambda \right) =\sigma _{abs}\left( \lambda \right) + \sigma _{coh}\left( \lambda \right) + \sigma _{inc}\left( \lambda \right) + \sigma _{inel}\left( \lambda \right) \end{aligned}$$

The microscopic cross-sections were calculated using the Monte-Carlo based simulation tool NCrystal^20–22^. The linear wavelength dependence of the absorption cross-sections is calculated using the 1/*v* model using the elemental composition and the densities listed in 3. The crystallography data for h-BN was taken from^39^ (Crystallography Open Database ID (COD ID) 9008997^40–46^) and the data for ${\hbox {B}_{4}}\hbox {C}$ from^47^ (COD ID 4124697). The amorphous plastics PLA and PA were modeled using AFGA^17^.

**Figure 10:**
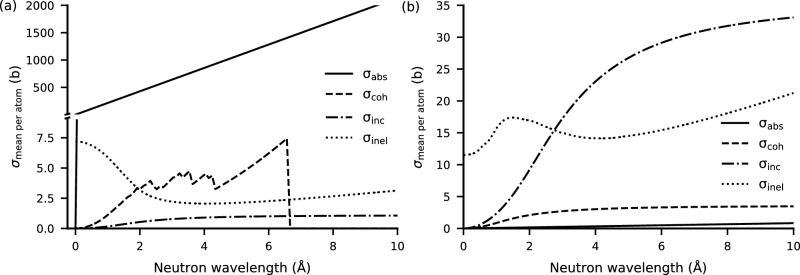
Microscopic cross-sections for neutron interaction with h-BN (**a**) and PLA (**b**) depending on the neutron wavelength. The cross-sections for absorption (solid) and coherent-elastic (dashed), incoherent-elastic (dash-dotted), and inelastic (dotted) scattering cross-sections were calculated using NCrystal. The material properties used are shown in Table 3, and the crystallographic data for h-BN was taken from Wyckoff et al.^39^. PLA was modelled using AFGA^17^.

The resulting microscopic wavelength-dependent cross-sections for h-BN are shown in Fig. 10a. The absorption cross-section $\sigma _{abs}$ (solid line) increases strongly and linearly with increasing wavelength. The coherent elastic cross-section $\sigma _{coh}$ (dashed line) shows characteristic Bragg-edges with sharp dips in cross-section at the wavelengths corresponding to backscattering on the lattice planes present in the crystal structure. Therefore, for wavelengths longer than the maximum d-spacing in h-BN $\sigma _{coh}$ vanishes. The incoherent elastic $\sigma _{inc}$ (dash-dotted line) approaches zero for short wavelengths and levels off to a low, approximately constant value for long wavelengths. The inelastic scattering cross-section $\sigma _{inel}$ (dotted line) is in the same order of magnitude as $\sigma _{coh}$ but dips from 7.5 $\hbox {b}$ at short wavelengths to 2.5 $\hbox {b}$ between 2 Å to 4 Å and slowly rises towards long wavelengths.

Figure 10b shows the microscopic cross-sections for PLA. The main contribution to the total cross-section is from the incoherent scattering (dash-dotted line), which increases towards long wavelengths while leveling off to approximately 35 $\hbox {b}$. The inelastic scattering cross-section (dotted line) ranges between approximately 11 $\hbox {b}$ to 20 $\hbox {b}$ over the given wavelength range with a local maximum at approximately 1.5 Å followed by a local minimum around 4 Å and a subsequent steady slow rise towards long wavelengths. The coherent elastic scattering cross-section (dashed line) lacks Bragg-edges due to the missing ordering in the amorphous material. The absorption cross-section (solid line) and the coherent elastic cross-section, are below 3 $\hbox {b}$ across the wavelength range.

The main contribution to the neutron attenuation of the mixed materials is the Boron filler, followed by the incoherent elastic scattering of the Hydrogen-rich plastic.

**Figure 11:**
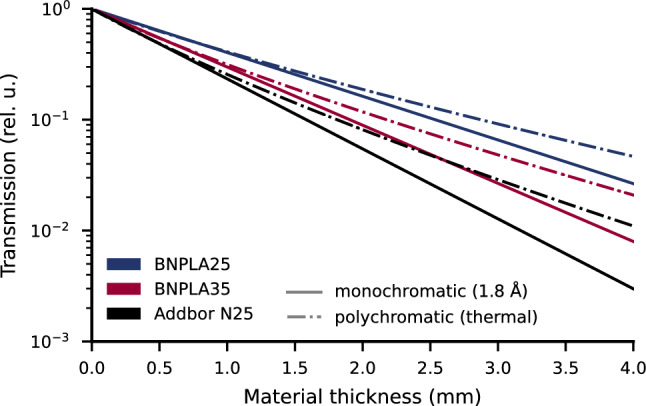
Expected neutron transmission over material thickness for a neutron wavelength of 1.8 Å (solid lines) and a simple Maxwellian thermal spectrum with its peak at 1.8 Å (dash-dotted lines) resulting from the microscopic cross-sections for different shielding material mixtures. The neutron transmission for a monochromatic neutron beam decreases purely exponentially (linearly on a logarithmic scale) with the material thickness. The slope of the lines steepens with increasing Boron content, from BNPLA25 (dark blue) to BNPLA35 (red) and Addbor N25 (black). The transmission in a polychromatic beam shows an increasing deviation from a single exponential decay towards longer wavelengths due to beam-hardening.

By summation of the different cross-sections (Eq. 3), the total wavelength-dependent microscopic cross-section of a single material was calculated. The material mixtures were implemented using the $\texttt {``phases<>''}$ syntax with the volume mixing ratios given in Tables 3 and 4. In the subsequent calculation of the linear attenuation coefficient (Eq. 2), the attenuation of the material mixtures was calculated from the densities of the single material components (see Table 3) and their mass fractions in the mixture as noted in Table 4. The transmission of the material mixtures was then obtained from Eq. 1. The resulting expected neutron transmission at 1.8 Å through a given thickness of the shielding material compositions BNPLA25, BNPLA35, and Addbor N25 is shown in Fig. 11. The transmission decreases exponentially with increasing thickness (linear in logarithmic scale) for a monochromatic neutron beam of 1.8 Å (solid lines). The different material compositions show higher attenuation per material thickness with increasing boron content from BNPLA25 with 25 %wt h-BN (blue), BNPLA35 with 35 %wt h-BN (red) to Addbor N25 (25 %wt ${\hbox {B}_{4}}\hbox {C}$) (black). In the more realistic case of a polychromatic thermal neutron spectrum, the wavelength dependence of the cross-sections causes a deviation from the pure exponential decay. The increase in absorption cross-section towards long wavelengths leads to long wavelengths being absorbed disproportionally stronger than short wavelengths. This causes the spectrum of the neutron beam to shift towards shorter wavelengths over the thickness of the material, and the resulting hardened spectrum causes the neutron beam to not be attenuated as strongly as the initial beam with a softer spectrum. Effectively leading to higher transmission than expected, especially for thick and strongly absorbing samples. This effect is generally referred to as beam-hardening and can be calculated by integrating over the contribution of each wavelength to the total transmission^48^.

4 $$\begin{aligned} T = 1/\phi _{total} \int _{\lambda _{min}}^{\lambda _{max}} \phi \left( \lambda \right) T\left( \lambda \right) d\lambda , \end{aligned}$$

with $\phi$ being the neutron flux at a given wavelength $\lambda$ and $\phi _{total}$ being the total integral neutron flux. By assuming a simple Maxwellian spectrum with its peak at 1.8 Å and integrating the calculated wavelength-dependent transmission (Eq. 1) according to Eq. 4 for each thickness, the dotted lines in Fig. 11 were obtained. The resulting transmission of a polychromatic thermal spectrum is generally higher than the transmission of a 1.8 Å monochromatic neutron beam. The deviation from the transmission of a monochromatic beam is higher with increasing thickness and boron content, depicting the expected effects of beam-hardening.

The bulk transmission of materials consisting of strongly absorbing particles in less absorbing matrix material can be higher than expected due to channeling effects as described by Burrus^49^. This is especially relevant for large particles at low volume fractions. BNPLA, however, is expected to show negligible channeling effects due to the small and polydisperse particle distribution of h-BN combined with the relatively high concentration.

**Table 3:** Densities and chemical composition of the raw materials used to calculate neutron cross-sections of the composite shielding materials.

Material	Chemical composition	Density ($\hbox {g}\,\hbox {cm}^{-3}$)	COD ID	AFGA
PLA	${[\hbox {C}_{3}\hbox {H}_{4}\hbox {O}_{2}]_{n}}$	1.24	–	$\texttt{1xCH3+1xCHali}$
PA (6.6)	${[\hbox {C}_{12}\hbox {H}_{22}\hbox {N}_{2}\hbox {O}_{2}]_{n}}$	1.15	–	$\texttt{10xCH2+2xNH}$
h-BN	BN	2.1	9008997	–
${\hbox {B}_{4}}\hbox {C}$	${\hbox {B}_{4}}\hbox {C}$	2.52	4124697	–

**Table 4:** Densities and mass fractions of the raw materials used to calculate neutron linear attenuation coefficients of the composite shielding materials.

Material	Nominal density ($\hbox {g}\,\hbox {cm}^{-3}$)	Mixing ratio	
		By weight	By volume
BNPLA25	1.38	25 %wt h-BN, 75 %wt PLA	16.4 %vol h-BN, 83.6 %vol PLA
BNPLA35	1.45	35 %wt h-BN, 65 %wt PLA	24.1 %vol h-BN, 75.9 %vol PLA
Addbor N25	1.32	25 %wt ${\hbox {B}_{4}}\hbox {C}$, 75 %wt PA	13.2 %vol ${\hbox {B}_{4}}\hbox {C}$, 86.8 %vol PA

Nominal density and mixing ratio by volume calculated from mixing ratio by weight and densities of the raw materials given in Table 3.

### Neutron transmission measurements

**Figure 12:**
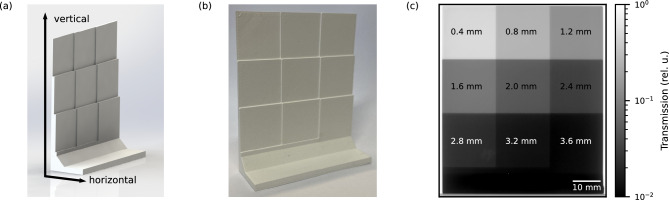
(**a**) Render of the 3D model used as step-wedge sample with the different build directions indicated by arrows with the layers oriented perpendicular to the respective direction. (**b**) Photograph of B25h, a printed step-wedge sample printed in horizontal build direction. (**c**) Neutron transmission scaled logarithmically of a printed step-wedge (B35h). The thickness of the material increases in 0.4 $\hbox {mm}$ steps from top left (0.4 $\hbox {mm}$) to bottom right (3.6$\,\hbox {mm}$) as marked in the transmission map.

To assess the shielding properties and verify the calculated neutron attenuation of the material as well as investigate the influence of printing parameters on the shielding properties, step-wedges with varying thickness (see Fig. 12a) were printed. Each step-wedge sample has nine different thicknesses over an area of each 20x20 $\hbox {mm}^{2}$. The thickness increment of the step-wedges was chosen to match the printer nozzle diameter of 0.4 $\hbox {mm}$, as shown in Fig. 12c. This ensures an accurate thickness of the steps independent of the orientation of the build direction. Several step-wedges were printed with different materials and printing parameters. One sample each was printed in horizontal build direction using BNPLA25, in the following called B25h, BNPLA35 (B35h) and Addbor N25 (A25h). A photograph of B25h is shown in Fig. 12b. The build directions and a render of the step-wedge are shown in Fig 12a. An additional sample was printed from BNPLA25 in vertical build direction (B25v). Finally, a step-wedge for each build direction was printed from BNPLA25 with not optimized printing parameters leading to small gaps in the printed volume, B25h-no in horizontal and B25v-no in vertical build direction. In addition to the printed step-wedges, two blocks with 8 $\hbox {mm}$ thickness were produced by smelting and casting in an Aluminum mold. One from BNPLA25 (B25c) and the second one from pure PLA (PLAc). A summary of the samples used for transmission measurements can be found in Table 5.

**Table 5:** List of samples used for neutron transmission measurements.

Sample name	Material	Manufacturing
B25v	BNPLA25	Step-wedge printed vertically
B25v-no	BNPLA25	Step-wedge printed vertically; not optimized
B25h	BNPLA25	Step-wedge printed horizontally
B25h-no	BNPLA25	Step-wedge printed horizontally; not optimized
B35h	BNPLA35	Step-wedge printed horizontally
A25h	Addbor N25	Step-wedge printed horizontally
B25c	BNPLA25	Cast block
PLAc	PLA	Cast block

We performed spatially resolved neutron transmission measurements at the imaging beamline NeXT at ILL using a polychromatic cold neutron beam^15^. The detector consisting of a Hamamatsu Orca Flash 3.0 sCMOS camera and 50 $\upmu \hbox {m}$ LiF:ZnS scintillation screen in typical configuration for neutron imaging was positioned at ${10}\,\hbox {m}$ distance from a 30 $\hbox {mm}$ pinhole.

The transmission of each of the step-wedges printed from BNPLA25 and Addbor N25 (B25v, B25h, A25h) was measured directly in front as well as at a distance of 150 $\hbox {mm}$ from the scintillation screen. Placing the sample further from the detector reduces the spatial resolution and thus blurs the resulting image, but also reduces the amount of scattered neutrons hitting the detector, leading to a more precise measurement of the transmission. The step-wedge printed from BNPLA35 (B35h) was placed directly in front of the detector. For each step-wedge, ten acquisitions of each 1$\,\hbox {s}$ exposure time were taken and subsequently combined with a pixel-wise median to remove outliers introduced by gamma radiation hitting the detector. The transmission maps, such as shown in Fig. 12c for B35h, were generated by referencing the images to open beam and dark images. Averaging the transmission values over the area of a single step yields the transmission values for each thickness.

### ToF transmission and SANS measurements

Both the wavelength-resolved ToF transmission data, and the SANS data, were taken at the D33 beamline of ILL^16^. For this, four discs with a diameter of 25 $\hbox {mm}$ and different thicknesses were printed from BNPLA35. The actual thicknesses over the area of the approximately 20 $\hbox {mm}$ diameter beamspot (Table 6) were verified with a micrometer. The wavelength-dependent linear attenuation coefficient $\Sigma ({\lambda })$ of BNPLA35 was calculated using the Beer–Lambert law. From Eq. 1 follows:

5 $$\begin{aligned} \Sigma (\lambda ) = ln(-T(\lambda )/t), \end{aligned}$$

enabling the calculation of $\Sigma (\lambda )$ from transmission $T(\lambda )$ independent of thickness *t*.

**Table 6:** Thickness of the four samples for SANS measurements over the area covered by the beamspot.

Sample	Thickness (mm)
#0	$0.20^{+2}_{-1}$
#1	$0.30^{+4}_{-1}$
#2	$0.40^{+2}_{-21}$
#3	$0.50^{+5}_{-2}$

### Manufacturing of material and samples

BNPLA25 and BNPLA35 were manufactured by colorFabb BV^50^ as two separate batches. The h-BN was purchased at Henze Boron Nitride Products AG in the form of a lubricant additive with 3.5 $\upmu \hbox {m}$ particle size called HeBoFill® BL-SP 035^23^. BNPLA25 was compounded in a batch of 50 $\hbox {kg}$ with a content of 12.5 $\hbox {kg}$ h-BN resulting in a mixture with 25 %wt h-BN content. The second batch was started with 25 kg (50 %wt) h-BN but had to be thinned to 35 %wt h-BN (BNPLA35) content, as the high filler content resulted in the filament being too brittle to be coiled on spools, resulting in BNPLA35.

Both BNPLA25 and BNPLA35 show very similar behavior while printing and can be printed with the default factory presets for regular PLA. For ideal results, it might be necessary to fine-tune some parameters depending on the printer and preset. BNPLA35 filament is relatively brittle due to the high h-BN content and tends to break occasionally if cold. We, therefore, recommend a printer enclosure to keep a higher (ca. ${30}^{\circ }\hbox {C}$) ambient temperature and a filament run-out sensor to automatically pause the print if the filament breaks. BNPLA25 however, showed no such issues in our testing. From our experience the printed parts show similar mechanical properties as regular PLA, which is illustrated as well with the identical tensile strength.

The samples used for the transmission measurements listed in Table 5 were printed with a Prusa i3 MK3^51^ and a Leapfrog Bolt Pro^52^. In general the factory print settings for PLA gave very similar print results for regular PLA as for BNPLA. The Leapfrog settings required, in general, significantly more adjustment for optimal results compared to the Prusa settings both for PLA and for BNPLA. All samples except the not optimized (B25h-no and B25v-no) were printed with a Prusa i3 MK3. This was done to clearly show the effects of not optimized print settings.

The samples for tensile testing and the samples for measurements at D33 (SANS and ToF transmission) were printed with a Prusa i3 MK3 and optimized print settings.

## Data Availability

The data is available from the corresponding author upon reasonable request.
